# Cognitive impairment associated with psychosis (CIAPS): Validity of clinical criteria to detect cognitive impairment

**DOI:** 10.1192/j.eurpsy.2021.1390

**Published:** 2021-08-13

**Authors:** A. Sánchez-Torres, G. Gil-Berrozpe, R. Lorente-Omeñaca, M. Zandio, L. Moreno-Izco, L. Janda, D. Peralta, V. Peralta, M. Cuesta

**Affiliations:** 1 Mental Health Group, Instituto de Investigación Sanitaria de Navarra (IdISNA), Pamplona, Spain; 2 Psychiatry, Complejo Hospitalario de Navarra, Pamplona, Spain; 3 Mental Health Department, Servicio Navarro de Salud-Osasunbidea, Pamplona, Spain

**Keywords:** psychosis, schizophrénia, cognition, diagnostic criteria

## Abstract

**Introduction:**

Even though cognitive impairment is considered a hallmark of schizophrenia, it has not been included as a criterion into major diagnostic systems.

**Objectives:**

To test whether a set of clinical-defined cognitive impairment criteria can have utility in the assessment of psychosis patients in clinical practice.

**Methods:**

We assessed 98 patients with a psychotic disorder, diagnosed using DSM 5 criteria. We developed a set of cognitive impairment associated with psychosis (CIAPs) criteria following the format of current DSM criteria and based on previous literature. The CIAPs criteria include: A) criterion for evidence of cognitive impairment after the beginning of illness; B) cognitive impairment clinically evidenced, affecting functioning in everyday activities in at least two out of six cognitive domains; C) and D) exclusion criterion for either delirium or other neurocognitive disorders, respectively, as causal agents of the cognitive impairment. The psychosis patients dichotomized by the CIAPs criteria were tested regarding the neuropsychological performance in attention, speed of processing, verbal memory, visual memory, working memory, executive function and social cognition tasks. Also a Global Cognitive Index was calculated.

**Results:**

Forty-three patients with psychosis fulfilled the CIAPs criteria (43.9%). MANOVA profile analyses revealed a pattern of statistically significant deficits in all the cognitive dimensions except for social cognition in CIAPs+ patients regarding CIAPS-, with prominent deficits in processing speed and memory functions.

**Figure:**
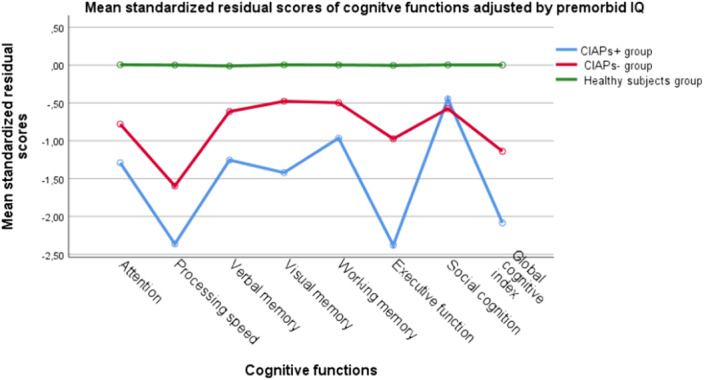

**Conclusions:**

The CIAPs criteria could be an auxiliary method for clinicians to assess cognitive impairment. It may also permit clinical estimation of the influence of cognitive deficits on the ecological functioning of patients.

**Conflict of interest:**

This work was supported by the Government of Navarra (grants 17/31, 18/41, 87/2014) and the Carlos III Health Institute (FEDER Funds) from the Spanish Ministry of Economy and Competitivity (14/01621 and 16/02148). Both had no further role in the study des

